# Free Polyethylenimine Enhances Substrate-Mediated Gene Delivery on Titanium Substrates Modified With RGD-Functionalized Poly(acrylic acid) Brushes

**DOI:** 10.3389/fchem.2019.00051

**Published:** 2019-02-07

**Authors:** Amy Mantz, Alice Rosenthal, Eric Farris, Tyler Kozisek, Eva Bittrich, Saghar Nazari, Eva Schubert, Mathias Schubert, Manfred Stamm, Petra Uhlmann, Angela K. Pannier

**Affiliations:** ^1^Department of Biological Systems Engineering, University of Nebraska-Lincoln, Lincoln, NE, United States; ^2^Center for Nanohybrid Functional Materials, University of Nebraska-Lincoln, Lincoln, NE, United States; ^3^Leibniz-Institut für Polymerforschung Dresden e.V., Dresden, Germany; ^4^Institute of Polymeric Materials, Technische Universität Dresden, Dresden, Germany; ^5^Department of Electrical and Computer Engineering, University of Nebraska-Lincoln, Lincoln, NE, United States; ^6^Department of Physics, Chemistry, and Biology, Linkoping University, Linkoping, Sweden; ^7^Terahertz Materials Analysis Center (THeMAC), Linkoping University, Linkoping, Sweden; ^8^Department of Chemistry, University of Nebraska-Lincoln, Lincoln, NE, United States

**Keywords:** substrate mediated, polymer brushes, poly(acrylic) acid, nonviral gene delivery, branched polyethylenimine, RGD ligand

## Abstract

Substrate mediated gene delivery (SMD) is a method of immobilizing DNA complexes to a substrate via covalent attachment or nonspecific adsorption, which allows for increased transgene expression with less DNA compared to traditional bolus delivery. It may also increase cells receptivity to transfection via cell-material interactions. Substrate modifications with poly(acrylic) acid (PAA) brushes may improve SMD by enhancing substrate interactions with DNA complexes via tailored surface chemistry and increasing cellular adhesion via moieties covalently bound to the brushes. Previously, we described a simple method to graft PAA brushes to Ti and further demonstrated conjugation of cell adhesion peptides (i.e., RGD) to the PAA brushes to improve biocompatibility. The objective of this work was to investigate the ability of Ti substrates modified with PAA-RGD brushes (PAA-RGD) to immobilize complexes composed of branched polyethyleneimine and DNA plasmids (bPEI-DNA) and support SMD in NIH/3T3 fibroblasts. Transfection in NIH/3T3 cells cultured on bPEI-DNA complexes immobilized onto PAA-RGD substrates was measured and compared to transfection in cells cultured on control surfaces with immobilized complexes including Flat Ti, PAA brushes modified with a control peptide (RGE), and unmodified PAA. Transfection was two-fold higher in cells cultured on PAA-RGD compared to those cultured on all control substrates. While DNA immobilization measured with radiolabeled DNA indicated that all substrates (PAA-RGD, unmodified PAA, Flat Ti) contained nearly equivalent amounts of loaded DNA, ellipsometric measurements showed that more total mass (i.e., DNA and bPEI, both complexed and free) was immobilized to PAA and PAA-RGD compared to Flat Ti. The increase in adsorbed mass may be attributed to free bPEI, which has been shown to improve transfection. Further transfection investigations showed that removing free bPEI from the immobilized complexes decreased SMD transfection and negated any differences in transfection success between cells cultured on PAA-RGD and on control substrates, suggesting that free bPEI may be beneficial for SMD in cells cultured on bPEI-DNA complexes immobilized on PAA-RGD grafted to Ti. This work demonstrates that substrate modification with PAA-RGD is a feasible method to enhance SMD outcomes on Ti and may be used for future applications such as tissue engineering, gene therapy, and diagnostics.

## Introduction

Nonviral gene delivery is the delivery of exogenous genetic material to cells or tissues, generally to produce a therapeutic protein, with applications in gene therapy, tissue engineering and regenerative medicine, and biomedical implants. Nonviral gene delivery is often performed using cationic polymer or lipid vectors complexed with DNA plasmids through electrostatic interactions. The formed complexes are typically delivered using a bolus method, which can be limited by mass transport to the cells and leaves the complexes susceptible to processes such as degradation and aggregation, thereby limiting gene transfer (Al-Dosari and Gao, 2009). Substrate-mediated gene delivery (SMD), also known as reverse transfection or solid-phase delivery, is a method of immobilizing DNA complexes to the substrate via covalent attachment or nonspecific adsorption. Compared to bolus delivery, SMD has been shown to limit complex aggregation and require a lower dose of DNA, as well as increase transgene expression and the number of transfected cells by increasing the local concentration of DNA within the microenvironment around the cell and overcoming a mass transport barrier to gene delivery efficiency (Pannier and Shea, 2004; Bengali et al., 2005, 2007; Pannier et al., 2005, 2008; Rea et al., 2009a; Wang et al., 2010; Pannier and Segura, 2013). Although a promising delivery method, past investigations into SMD have focused on using tissue engineering scaffolds like poly(lactide-co-glycolide) (PLG) (Shea et al., 1999; Jang et al., 2005) or traditional culturing substrates such as tissue culture polystyrene (TCPS), with or without protein coatings (Bengali et al., 2005, 2009; Rea et al., 2009b), but few SMD studies have focused on the modification of commonly used metal biomaterials (Zhang et al., 2015; Shekhar et al., 2018). For example titanium (Ti) is one of the most commonly used biomaterials (Elias et al., 2008), with many applications that could benefit from nonviral SMD such as enhancing the integration of bone implants by delivering genes to increase osseointegration (Wang et al., 2015; Zhang et al., 2015), gene-eluting stents to accelerate re-endothelialization (Sharif et al., 2012), or developing implantable sensors protected by the local delivery of anti-inflammatory and anti-fibrosis genes (Klueh et al., 2014), but to date there have been few studies published using SMD on Ti.

Along with the limited scope of biomaterials investigated for nonviral SMD, further tunings of the substrate to enhance DNA complex interactions and cell-material interactions are necessary to make SMD more efficient and therapeutically relevant. Polymer brushes are an attractive substrate modification for SMD, as the brushes have stimuli-responsive and bioactive properties (Krishnamoorthy et al., 2014; König et al., 2018), can be engineered for controlled cellular response through covalent binding of adhesions peptides (Psarra et al., 2015a, 2017; Alas et al., 2017; Rosenthal et al., 2018), and can be used to control the adsorption of proteins and release of biomolecules (Dai et al., 2006; Hollmann and Czeslik, 2006; Chiang et al., 2011; Psarra et al., 2015a; Bittrich et al., 2018). Polymer brushes are formed by grafting polymer chains adjacently on a substrate, which forces the chains to stretch from the substrate (Milner, 1991; Brittain and Minko, 2007). There are two common approaches for grafting polymer brushes, “grafting from” and “grafting to.” For the “grafting from” approach, a substrate is modified with initiator sites and then exposed to monomers, which are polymerized on the surface, often by radical polymerization strategies. In this “grafting from” approach, homogenous brushes are formed with high brush density but are more difficult to produce and characterize (Zdyrko and Luzinov, 2011). For the “grafting to” method, polymer chains are formed before grafting to the substrate and added to the surface via chemical reactions between reactive groups on the surface and a functional end group of the polymer (Zhao and Brittain, 2000). With the “grafting to” approach, “pseudo”-brushes with more than one grafting point per chain can be prepared with swelling properties not distinguishable from end-grafted brushes (Aulich et al., 2010; Bittrich et al., 2010). Although less dense compared to “grafting from” brushes, the “grafting to” approach, in general, produces homogeneous polymer brushes with a well-defined structure and higher stability compared to physically adsorbed polymers (Minko, 2006; Zdyrko and Luzinov, 2011; Li and Sheiko, 2015).

**Figure 1:**
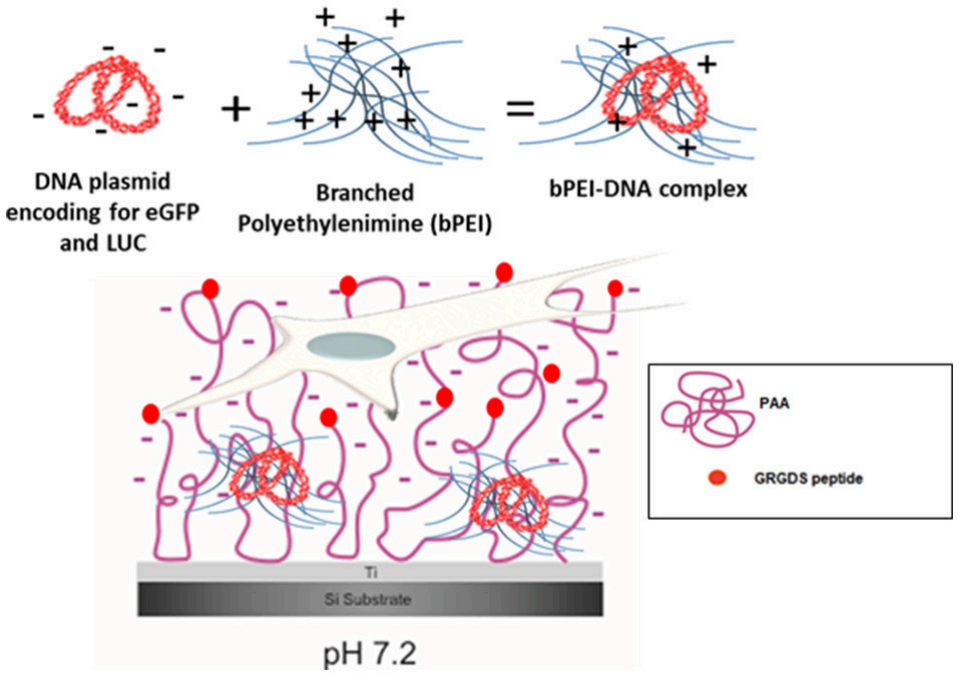
bPEI-DNA complex immobilization on PAA brushes at pH 7.2. Complex formation with DNA plasmid encoding for enhanced green fluorescent protein (eGFP) and luciferase (LUC) and branched polyethylenimine (bPEI) at a N/P ratio of 20 complexes with an overall positive charge (6 mV). These positively charged bPEI-DNA complexes can interact with negatively charged, swollen PAA-RGD brushes (pH 7.2) on the substrate to transfect NIH/3T3 fibroblasts cultured on the substrate.

While methods to produce polymer brushes on silicon and other materials (i.e., gold, stainless steel) are well-known (Callewaert et al., 2005; Wu et al., 2007; Bittrich et al., 2010; Chiang et al., 2011; Kasputis et al., 2013; Akkilic et al., 2016), in our recent paper (Rosenthal et al., 2018), we showed for the first time that the poly(acrylic) acid (PAA) brush “grafting to” process is feasible on Ti substrates and the pH-responsive deprotonation of the PAA brushes is maintained. Furthermore, following the addition of the RGD-containing peptide GRGDS to the brushes (PAA-RGD), cell adhesion of NIH/3T3 fibroblasts was significantly enhanced compared to cells cultured on unmodified PAA brushes. Swollen deprotonated brushes have been shown to produce negatively charged polymer chains at a pH of 7.2 (Psarra et al., 2015b), and that charge was further decreased by the inclusion of RGD peptides (Psarra et al., 2017). Therefore, given the negative charge of the PAA brushes and the inclusion of the RGD peptide (Figure 1), we propose that Ti substrates modified with PAA-RGD are an ideal platform for SMD, as PAA brushes could improve the loading of cationic DNA complexes through charge interactions and mediate cell adhesion via the RGD peptide. In this work we expand on our previous study by showing, for the first time, the feasibility of immobilizing complexes formed with branched polyethylenimine and DNA plasmids (bPEI-DNA) onto PAA-RGD brushes (Figure 1), and characterize their release and transfection ability, as well as propose a potential benefit of PAA-RGD brushes to allow for the presentation of free bPEI to cells to improve gene delivery.

## Materials and Methods

### Preparation of PAA Brushes on Ti Surface and Covalent Bonding of RGD/RGE Peptides

Throughout this study the substrates investigated include PAA brushes on Ti (abbreviated as PAA), PAA brushes modified with GRGDS on Ti (abbreviated as PAA-RGD), PAA brushes modified with the control peptide RGES on Ti (abbreviated as PAA-RGE), and Ti with no modification (termed Flat Ti) as a control. Ti substrates (100 nm Ti, Grade 2, on a Si wafer) were purchased from Platypus Technologies (Madison, WI) and used for flat controls. Ti substrates for polymer brush functionalization were produced by Fraunhofer IWS (Dresden, DE) by sputtering Ti pellets (Grade 2) on Si wafer (Silicon Materials, Germany) or fabricated in an ultra-high deposition vacuum chamber by electron beam evaporation of Ti pellets (Super Conductor Materials, Inc., Tallman, NY) onto Si wafer substrates (University Wafer, South Boston, MA) (Rosenthal et al., 2018). Samples were functionalized with polymer brushes according to our previously reported “grafting-to” method (Rosenthal et al., 2018). Briefly, the Ti substrate was activated with oxygen plasma for 1 min (Plasma Cleaner PDC-002 with Plasmaflo PDC-FMG-2, Harrick Plasma, USA). After activation, a solution composed of 0.02 wt % of poly(glycidyl) methacrylate (PGMA, Mn = 17,500 g/mol, Mw/Mn = 1.7, Polymer Source,Inc., Canada) in chloroform (CHCl_3_, Fisher Scientific, UK) was spin-coated (Spin150 spin coater, Polos, Putten, Netherlands). The PGMA layer was annealed for 10 min at 110°C under vacuum, resulting in a thin reactive anchoring layer with epoxy groups for the adjacent grafting step. A PAA (Mn = 26,000 g/mol, Mw/Mn = 1.12, Polymer Source, Inc., Canada) solution was prepared at 1.0 wt % in ethanol (EtOH) and spin-coated onto the grafted PGMA layer. The PAA layer was annealed at 80°C for 30 min under vacuum to react to the epoxy groups of PGMA with COOH groups along the chain of PAA, grafting the PAA chains in loops and tails via ester bonds. Excess polymer was extracted by stirring the samples in ethanol for 30 min at room temperature and dried with a N_2_ flux. Peptide conjugation based on the carboxyl-amine-reaction (EDC-NHS coupling) was performed as previously described (Rosenthal et al., 2018) and all materials used for peptide conjugation were purchased from Sigma-Aldrich (St. Louis, MO). Briefly, buffers were prepared using boric acid, phosphate-buffered saline (PBS), and 2-(*N*-morpholino)ethanesulfonic acid (MES). The linear RGD-containing peptide GRGDS (or RGE-containing peptide RGES) was covalently bound to PAA brushes on Ti surfaces via activation of the PAA carboxy groups with *N*-(3-dimethylaminopropyl)-*N*′-ethylcarbodiimide hydrochloride (EDC) and *N*-hydroxysuccinimide (NHS) for direct conjugation of carboxyl groups with the primary amines of the peptides. For conjugation, PAA brushes on Ti substrates were equilibrated in 0.1 M MES at pH 6 for 10 min. After aspiration of this buffer, brushes were reacted with 0.5 mL of 5 mM EDC solution and 0.5 mL of 2 mM NHS solution in 0.1 M MES buffer (pH 6) by gently shaking for 40 min. Subsequently, a 1.0 mg/mL solution of GRGDS (or RGES) in 0.1 M borate buffer (pH 8) was added to the activated PAA brush substrates. After gentle shaking at room temperature for 16 h, the peptide solution was aspirated and the GRGDS (or RGES)-modified samples were washed three times by stirring in 0.1 M PBS buffer at pH 7.4 for 3 min.

### DNA Complex Formation and Characterization

Plasmid (pEGFP-LUC), that encodes both the enhanced green fluorescent protein (EGFP) and firefly luciferase protein (LUC) under the direction of a CMV promoter, was used in all studies in this work. Plasmids were purified from bacteria culture using Qiagen (Valencia, CA) reagents and stored in Tris–EDTA buffer solution (10 mM Tris, 1 mM EDTA, pH 7.4) at −20°C. For DNA complex formation, 25 kDa branched polyethylenimine (bPEI; Sigma-Aldrich) was dissolved in reduced serum medium OptiMEM (Fisher Scientific) and then added dropwise to DNA in OptiMEM, vortexed for 10 s, and incubated for 15 min at room temperature. Complexes were formed at nitrogen/phosphate (N/P) ratios of 3, 5, 10, or 20 in OptiMEM with 2 μg of DNA, and delivered in a volume of 3 mL for the spectroscopic ellipsometry measurement and 300 μL for all other studies, resulting in a DNA amount of 1 μg/cm^2^ used for immobilization to substrates in all studies.

The size and zeta potential of the bPEI/DNA complexes were determined by dynamic light scattering and Laser Doppler micro-electrophoresis, respectively, using a Zetasizer Nano ZS90 (Malvern Instruments Ltd, UK). Size measurements were taken at 25°C at a scattering angle of 90° and size reported as the Z-average diameter (d. nm). Zeta potential measurements were also taken at 25°C using folded capillary cells with the measurement mode set to automatic and the values reported in mV.

### Ellipsometric Measurements for Characterization of PAA Brushes and DNA Complex Immobilization

Ellipsometric measurements were acquired using a Woollam RC2 or a M2000-VI spectroscopic ellipsometer (both from J.A. Woollam, Co., Inc., Lincoln, NE, USA) to confirm brush parameters, as previously described (Rosenthal et al., 2018). Briefly, for dry brushes the ellipsometric data, Δ (relative phase shift) and tan Ψ (relative amplitude ratio), were recorded at wavelengths (λ) of 380–1,700 nm and four angles of incidence (AOI: 45, 55, 65, 75°). To confirm brush swelling and functionality (indicative of deprotonation), substrates were first sterilized with EtOH and then the pH-reactive brush swelling was performed by adding OptiMEM (pH 7.2) to dry PAA brushes. Brush swelling within OptiMEM was measured at AOI 70° with a batch cuvette (TSL Spectrosil, Hellma, Muellheim, Germany), at wavelengths λ = 400–1,200 nm. The brush film thickness was quantified via the change in Ψ and Δ, which was used to calculate the swelling degree (swollen brush thickness divided by dry brush thickness). Brush swelling was also measured before and after the addition of RGD and RGE peptides, as well as before and after complex immobilization, to determine the amount of peptide and complexes immobilized. These measurements were all performed *in situ*. Experimental data were modeled in CompleteEASE software (Version 4.64, J.A. Woollam Co., Inc., Lincoln, NE, U.S.A.) as described in our previous work (Rosenthal et al., 2018). The amounts of the peptides RGD and RGE at the PAA brush surface were calculated with a modified de Feijter approach (Equation 1) (König et al., 2018):

(1) $$\begin{array}{l} \Gamma_{peptide}{}_{(or\;complexes)}=d_{brush}\frac{n_{comb}-n_{brush}}{\left(\frac{d_{n}}{d_{c}}\right)}+d_{add}\frac{n_{comb}-n_{amb}}{\left(\frac{d_{n}}{d_{c}}\right)} \end{array}$$

In this approach, changes in the layer parameters *in-situ* refractive index and *in-situ* thickness (*n*_*comb*_*, d*_*comb*_) after covalent peptide immobilization are referenced to the swollen state of the surface (*n*_*brush*_*, d*_*brush*_) before immobilization, which are the parameters of the swollen PAA brushes (Equation 1). The amount of DNA complexes immobilized to the Flat Ti substrate was calculated by the de Feijter equation (De Feijter et al., 1978), while amounts of complexed DNA on PAA and PAA-RGD brushes were calculated again with the modified de Feijter approach (Equation 1), referencing the *in-situ* layer parameters (*n*_*comb*_*, d*_*comb*_) of the combined complexes and brushes to the parameters (*n*_*brush*_*, d*_*brush*_) of the swollen PAA brushes or the parameters of the swollen PAA-RGD brush, respectively. The refractive index increment *d*_n_/*d*_c_ = 0.185 cm^3^/g was used for the RGD peptides (Rosenthal et al., 2018) and *d*_n_/*d*_c_ = 0.183 cm^3^/g for the DNA complexes (Tumolo et al., 2004).

### DNA Complex Immobilization and Release Measured by Radiolabeled DNA

Plasmid radiolabeled with [α-^32^P]dATP (Perkin Elmer, Akron, OH) was used to measure the immobilization of DNA complexes on Flat Ti, PAA, and PAA-RGD substrates. To label the DNA plasmid, a nick translation kit (Invitrogen, Waltham, MA) was used following the manufacturer's protocol. The radiolabeled DNA was diluted with unlabeled DNA to a final concentration (0.806 μg/μL) and used to form DNA complexes, as described above. First, the substrates were prepared by cutting with a diamond-tipped scribe into pieces that fit into Falcon™ 48 well tissue culture plates (Fisher Scientific). Images of each substrate used for immobilization studies were taken prior to complex immobilization and analyzed with NIH ImageJ Processing Software to determine the surface area (cm^2^). Next, the substrates were bathed in 70% EtOH and then transferred to a new sterile well plate to air dry in a sterile biosafety cabinet. Complexes (300 μl in OptiMEM as described above) were immobilized by incubation on substrates for 2 h. After complex immobilization, the complex solution was removed and the substrates were washed twice with PBS. The quantity of DNA immobilized was determined by immersing substrates in a scintillation cocktail (5 mL, Thomas Scientific, Swedesboro, NJ) for measurement with a Packard Tri-Carb 1900 TR Liquid Scintillation Counter. Counts per minute were correlated to the DNA amount using a standard curve and the amount of DNA immobilized to each sample was normalized to the surface area (cm^2^).

The release profiles of immobilized DNA complexes from PAA, PAA-RGD, or Flat Ti were determined by incubation of the DNA-loaded substrates with either reduced serum OptiMEM, serum-containing cell growth media, or conditioned growth media (from flasks of NIH/3T3 fibroblasts cultured for 48 h) at 37°C in a humid chamber. At time 0, substrates with immobilized complexes were moved to a fresh well before adding the media to the substrates. At predetermined time points (0.5, 4, 24, and 48 h), the total volume of media was removed and counts per minutes were measured using a Packard Tri-Carb 1900 TR Liquid Scintillation Counter. An equal volume of fresh warmed media was then added to each substrate and the release was allowed to continue. At the final time point, the DNA remaining on the samples was also determined. The amount of DNA released from the substrate was determined from the measured counts per minutes using a standard curve with known amounts of DNA. The percentage of DNA released was calculated by dividing the cumulative counts released (at each time point) by the total counts initially on the substrate (determined by mass balance); thus, the release curves represent the percentage of DNA released relative to the initial amount bound to each surface.

### Cell Culture and Substrate Mediated Gene Delivery

Transfection studies were performed with murine fibroblast NIH/3T3 cells (ATCC, Manassas, VA) cultured in Dulbecco's Modified Eagle's Media (DMEM) completed with 10% Calf Serum (Colorado Serum Co., Denver, CO) and 1% Penicillin/Streptomycin. Fibroblasts were cultured at 37°C and 5% CO_2_ and passaged every 2 days with 0.05% Trypsin-EDTA. For transfection studies, substrates were cut and sterilized (as described above), bPEI-DNA complexes were formed and immobilized for 2 h onto the four substrate conditions (Flat Ti, PAA, PAA-RGD, and PAA-RGE), after which the solutions containing the DNA complexes were removed and then substrates were rinsed with OptiMEM before cells were seeded onto the substrates at a density of 50,000 cells/mL. Cells were cultured for 48 h at 37°C and 5% CO_2_ and then the substrates were transferred into a new well plate and lysed using 200 μL of 1X reporter lysis buffer (Promega, Madison, WI). As previously described in Hamann et al. (2018) and Kelly et al. (2016), transfection levels were quantified by measuring the luciferase activity using the Luciferase Assay System (Promega) and a luminometer (Turner Designs, Sunnyvale, CA). Luciferase activity (measured as relative light units, or RLUs) was normalized to the total protein amount determined with a Pierce BCA protein assay (Pierce, Rockford, IL), as seen in previous investigations.

### Cell Adhesion of NIH/3T3 Fibroblasts Cultured on PAA Brushes With Immobilized Complexes

To determine the effect of complex immobilization on the cellular response of NIH/3T3 fibroblasts cultured on bPEI-DNA complexes immobilized to PAA, PAA-RGD, PAA-RGE, and Flat Ti, calcein staining (Life Technologies, Carlsbad, CA) was used to visualize cellular adhesion and quantify the cell counts per area (cm^2^) at 48 h following cell seeding. Briefly, surfaces with adhered cells were transferred into new well plates prior to the assays. Substrates for staining were rinsed with PBS and then stained for 20 min in phenol-free DMEM (Fisher Scientific) with 2 μM Calcein-AM. Substrates were imaged with a Leica DMI 3000B fluorescence microscope (Leica Microsystems CMS GmbH, Wetzlar, Germany) and five images per well of three replicate wells were acquired using a 5x objective. Image analyses were performed using NIH ImageJ Processing Software to quantify cell counts.

### Assessing the Contribution of Free bPEI on Transfection Success With SMD

To assess the contribution of free bPEI on transfection success in NIH/3T3 fibroblasts cultured on bPEI-DNA complexes immobilized to PAA, PAA-RGD, PAA-RGE, and Flat Ti, complexes were first formed as previously described, and then filtered to remove free (uncomplexed) bPEI using a Vivaspin®6 Centrifugal Concentrator (Vivaproducts, Inc., Littleton, MA). Complexes were filtered by centrifuging the solution at 3,000 g for 3 min at 4°C. The DNA complexes trapped in the filter were eluted using an equal volume of OptiMEM. These filtered complexes were immobilized onto the substrates (PAA, PAA-RGD, PAA-RGE, and Flat Ti) and cells were cultured on these substrates and transfection was assessed, as described above.

To further understand the effect of free bPEI on transfection success in NIH/3T3 fibroblasts cultured on bPEI-DNA complexes immobilized to PAA, PAA-RGD, PAA-RGE, and Flat Ti, SMD transfection was performed with a controlled dosage of free bPEI. Filtered complexes formed as previously described received an addition of 1 or 5 μg of free bPEI during immobilization to the substrate, and then transfection was performed and assessed as described above.

### Cell Viability of NIH/3T3 Fibroblasts Cultured on PAA Brushes With Immobilized Filtered and Unfiltered Complexes

To understand the effect of immobilized complexes (and free bPEI) on the cellular response, the metabolic activity of cultured NIH/3T3 fibroblasts was assessed using a Water Soluble Tetrazolium (WST-1) salt cell proliferation assay kit (Roche, Indianapolis, IN), according to manufacturer's protocol, to quantify the cell viability at 48 h following cell seeding. Briefly, cells cultured on PAA-RGD, PAA-RGE, PAA, and Ti substrates (immobilized with unfiltered or filtered complexes) were transferred into new well plates prior to the assays. Cells were washed with 1 × PBS and incubated at 37°C in WST-1 solution (10 vol% WST-1 reagent in phenol-free Dulbecco's Modified Eagle Medium) for 3 h. After incubation, absorbance values were measured on an Epoch Microplate spectrophotometer (BioTek, Winooski, VT) at 430 nm and corrected with 690 nm as a reference wavelength, and then normalized per area (cm^2^).

### Statistical Analysis

All experiments were performed in triplicate on duplicate days, and values are reported from one representative experiment as means with standard error of the mean. Statistical comparisons were performed with Prism 5.0 graphing and statistical analysis software (Graph Pad, La Jolla, CA) at 95% confidence level (α = 0.05), with the statistical tests used specified in the figure legends.

## Results

### PAA Brush Film Characterization

The objective of this paper was to apply SMD to a Ti substrate functionalized with PAA brushes, further functionalized with RGD (or control RGE) peptides (Figure 1). First, the PAA brush parameters and pH swelling behavior were measured and modeled with spectroscopic ellipsometry to confirm the brush film thickness and swelling functionality of PAA brushes before the immobilization of bPEI-DNA complexes. Similar to our previous study where we functionalized Ti with PAA brushes (Rosenthal et al., 2018), the average film thickness for the activated oxide groups (*d*_*TiO*2_[nm]) after plasma activation, the PGMA anchoring layer (d_PGMA_ [nm]), and PAA brush thickness (*d*_PAA_ [nm]) were 0.8 ± 0.6, 1.9 ± 0.3, and 5.5 ± 0.3 nm, respectively (Table 1). After the addition of OptiMEM (pH 7.2; the reduced serum media used for complex immobilization), PAA brushes swelled to an average thickness of 23 ± 3.0 nm (average swelling degree of 4.0 ± 1.0, Table 2), which is similar to the swelling in 0.1 M PBS (pH 7.4) reported in our previous study (Rosenthal et al., 2018). Swelling measurements were also used to calculate RGD and RGE conjugation densities using Equation (1) (1.3 ± 0.2 and 1.0 ± 0.2 μg/cm^2^, respectively; Table 3), which is similar to the RGD density we reported in our previous study (Rosenthal et al., 2018).

**Table 1:** PAA brushes formed on Ti substrates.

**Replicate**	***d*_**TiO2**_ [nm]**	***d*_**PGMA**_ [nm]**	***d*_**PAA**_ [nm]**
1	0.2	2.3	5.1
2	0.7	1.8	5.7
3	1.4	1.7	5.6
Average	0.8 ± 0.6	1.9 ± 0.3	5.5 ± 0.3

The “grafting-to” process was monitored with spectroscopic ellipsometry at each step of the PAA brush formation. The first step is plasma activation for 1 min to form oxide groups (d_TiO2_ [nm]), and then a PGMA anchoring layer was spin-coated onto the activated substrate, and annealed at 110°C for 10 min under vacuum (d_PGMA_ [nm]). Next, a layer of PAA was spin-coated onto the PGMA anchoring layer and annealed at 80°C for 30 min under vacuum. Finally, the excess polymer was extracted with EtOH for 30 min at room temperature (d_PAA_ [nm]). Average values for each thickness are reported for three replicate substrates

**Table 2:** PAA brushes swelling in OptiMEM.

**Replicate number**	***d*_**PAA**_[nm] in cell**	***d*_**brush**_ in OptiMEM [nm]**	**Swelling degree**
1	5.2	26.0	5.0
2	6.7	23.2	3.4
3	6.3	19.7	3.1
Average	6.1 ± 0.8	23 ± 3.0	4.0 ± 1.0

*Swelling was performed to measure the increase in brush film thickness and calculate the swelling degree. The first measurement of the dry PAA brushes on Ti was performed in the cuvette (d_PAA_[nm] in cell). Next, the brushes were swollen by adding OptiMEM (pH 7.2) to PAA brushes **(**d_brush_ in OptiMEM [nm]). The swelling degree was calculated as a ratio of swollen thickness to dry thickness. Three replicate samples were measured and the average is given with the standard deviation of the data*.

**Table 3:** PAA Brushes with covalently bound peptide.

**Replicate**	***Γ*_RGD_[μg/cm^2^]**	***Γ*_RGE_[μg/cm^2^]**
1	1.5	1.0
2	1.2	1.2
3	1.2	0.9
Average	1.3 ± 0.2	1.0 ± 0.2

*Brush swelling of PAA brushes before and after covalent binding of GRGDS or RGES (c = 1 mg/ml) to PAA brushes were used to calculate the immobilized peptide amount (Γ_RGD/RGE_ [μg/cm^2^]) using a modified de Feijter approach. Three replicate samples were measured and the average is given with the standard deviation of the data*.

### Substrate Mediated Gene Delivery

**Figure 2:**
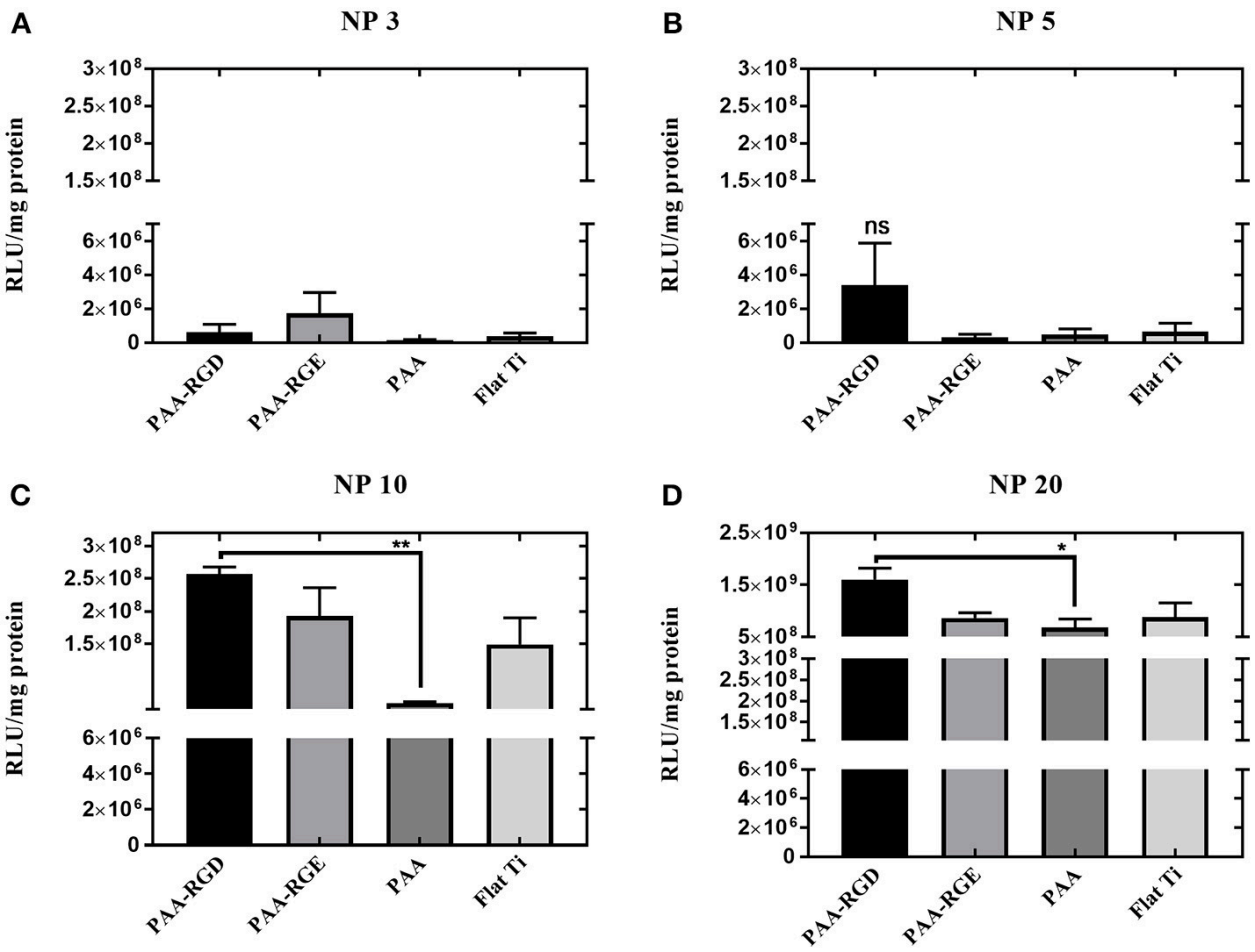
Substrate-mediated gene delivery of bPEI-DNA complexes in NIH/3T3 fibroblasts with varied N/P ratios. NIH**/**3T3 fibroblasts were cultured onto bPEI-DNA complexes formed with 2 μg of DNA, at N/P of 3, 5, 10, or 20, and immobilized to the substrate for transfection. SMD studies were analyzed using one-way ANOVA with Tukey's post-test, and cells cultured on complexes at N/P ratio of 3 or 5 has no statistical significance in transfection success for all substrates **(A,B)**, whereas cells cultured on immobilized complexes at N/P ratio of 10 showed a statistically significant increase in transfection of cells cultured on PAA-RGD compared to those cultured on PAA (^**^P ≤ 0.01) **(C)**, and cells cultured on immobilized complexes at N/P ratio of 20 had a statistically significant increase in transfection of cells cultured on PAA-RGD compared to those cultured on PAA (^*^P ≤ 0.05) **(D)**.

After assessing the brush formation and swelling behavior, the ability of the substrates modified with PAA brushes to support SMD was measured in NIH/3T3 fibroblasts and reported as transgene expression normalized to total amount of protein. Transfection was investigated as a function of the N/P ratio used to form bPEI-DNA complexes, which resulted in complexes with increasingly positive zeta potential and smaller diameter as the N/P ratio increased (Supplementary Figure 1), as expected. Transfection success increased for cells cultured on all substrates as the N/P ratio increased (Figure 2). Transfection with complexes formed at the lowest N/P ratio of 3 showed no significant difference in transfection success comparing all substrates (Figure 2A). While there was no significant difference in transfection measured in cells on all substrates with immobilized complexes formed at an N/P ratio of 5, transfection was increased by one order of magnitude for cells cultured on PAA-RGD compared to those cultured on PAA-RGE, PAA, and Flat Ti (Figure 2B). Finally, forming complexes at the higher N/P ratios of 10 and 20 resulted in a significant increase in transfection success (by up to an order of magnitude) in cells cultured on complexes immobilized to PAA-RGD compared to those cultured on PAA (Figure 2C; ^**^P ≤ 0.01; Figure 2D; ^*^P < 0.05). Given that complexes formed at the N/P ratio of 20 exhibited the highest transgene expression, further investigations on immobilization, release, and transfection were performed using this parameter.

### Immobilization and Release of DNA-bPEI Complexes

**Figure 3:**
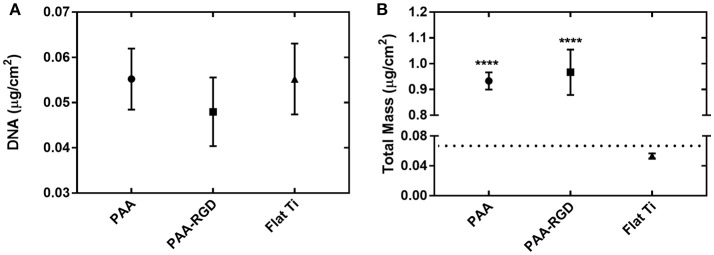
DNA complexes immobilized to PAA brushes compared to Flat Ti. For complexes formed at an N/P ratio of 20, the amount of material immobilized onto substrates measured by **(A)** radiolabeled DNA via scintillation counting and **(B)** total mass (bPEI and DNA plasmid, free and complexed) by spectroscopic ellipsometry. Statistical analyses were completed using one-way ANOVA with Tukey's post-test. There were no significant differences in the amount of DNA immobilized as measured by radioactivity **(A)**, but there were statistically significant differences between the amount of total mass on PAA-RGD and PAA substrates compared to Flat Ti (^****^P ≤ 0.0001) **(B)**. A dotted line marks the expected mass of bPEI-DNA complexes immobilized to the substrate based on the N/P ratio and quantification of DNA by radioactivity **(B)**.

To determine if the amount of DNA adsorbed into each substrate was the primary determinant for increased transfection success in cells cultured on PAA-RGD, the immobilization and release of bPEI-DNA complexes were analyzed. DNA complexes were loaded onto PAA, PAA-RGD, and Flat Ti substrates and the adsorbed amounts were measured by monitoring radiolabeled DNA plasmids with scintillation counting or total organic mass (bPEI, free and complexed to DNA, as well as DNA) with spectroscopic ellipsometry modeling. For immobilization determined with radioactivity, the amount of DNA adsorbed to PAA, PAA-RGD, and Flat Ti was 0.055 ± 0.007, 0.048 ± 0.008, and 0.055 ± 0.008 μg/cm^2^, respectively (Figure 3A); these amounts were not significantly different among the three substrates. For ellipsometry monitoring, the total mass of organic material adsorbed to the substrates was 0.93 ± 0.03 μg/cm^2^ for PAA, 0.97 ± 0.09 μg/cm^2^ for PAA-RGD, and 0.053 ± 0.003 μg/cm^2^ for Flat Ti, which showed a significant increase of adsorbed mass (i.e., DNA and free and complexed bPEI) on PAA-RGD and PAA compared to Flat Ti (Figure 3B; ^****^P ≤ 0.0001).

**Figure 4:**
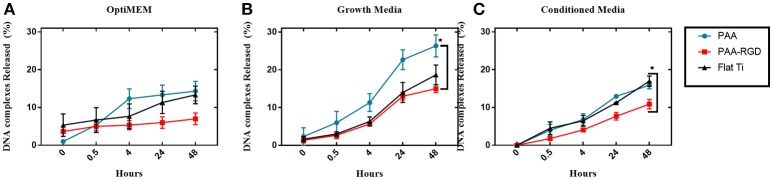
DNA complexes released from PAA and PAA-RGD brush substrates, compared to Flat Ti. The amount of DNA released from the substrates with OptiMEM **(A)**, serum-containing growth media **(B)**, or conditioned DMEM media **(C)** at 37°C was measured by radiolabeled DNA via scintillation counting. Release experiments were analyzed using one-way ANOVA with Tukey's post-tests at the final timepoint, which showed a statistically significant difference between PAA-RGD and PAA (^*^P ≤ 0.05) for release with growth media **(B)**, and a statistically significant difference between PAA-RGD compared to Flat Ti (^*^P ≤ 0.05) for release with conditioned media **(C)**.

To determine the effect of electrostatic and hydrophobic interactions between bPEI-DNA complexes and the substrates, and the contribution of complex release from substrates to transfection profiles, DNA release was quantified at multiple time points up to 48 h, using three different media: reduced serum OptiMEM, serum-containing cell growth media, or conditioned growth media (from flasks of cultured cells). The average percentages of total DNA released in OptiMEM from PAA-RGD, PAA, and Flat Ti after 48 h were 7.0 ± 1.5, 14 ± 2.6, and 13 ± 2.3%, respectively (Figure 4A), and there was no significant difference in the release of bPEI-DNA complexes from any of the substrates. The average percentage of total DNA released in serum-containing growth media for PAA-RGD, PAA, and Flat Ti at 48 h were 15 ± 1.0, 26 ± 2.9, and 19 ± 2.6%, respectively (Figure 4B), and the release of bPEI-DNA complexes from PAA substrates was significantly increased (11 ± 3.1%; ^*^P ≤ 0.05) compared to the release from PAA-RGD at the final time point when statistics were performed. Finally, the average percentage of total DNA released in conditioned growth media for PAA-RGD, PAA, and Flat Ti at 48 h were 11 ± 1.2, 16 ± 1.0, and 17 ± 1.3%, respectively (Figure 4C) and the release of bPEI-DNA complexes from Flat Ti was significantly increased (5.0 ± 1.6%; ^*^P ≤ 0.05) compared to the release from PAA-RGD.

### Cellular Adhesion and Viability on DNA-bPEI Complexes Immobilized on Substrates

**Figure 5:**
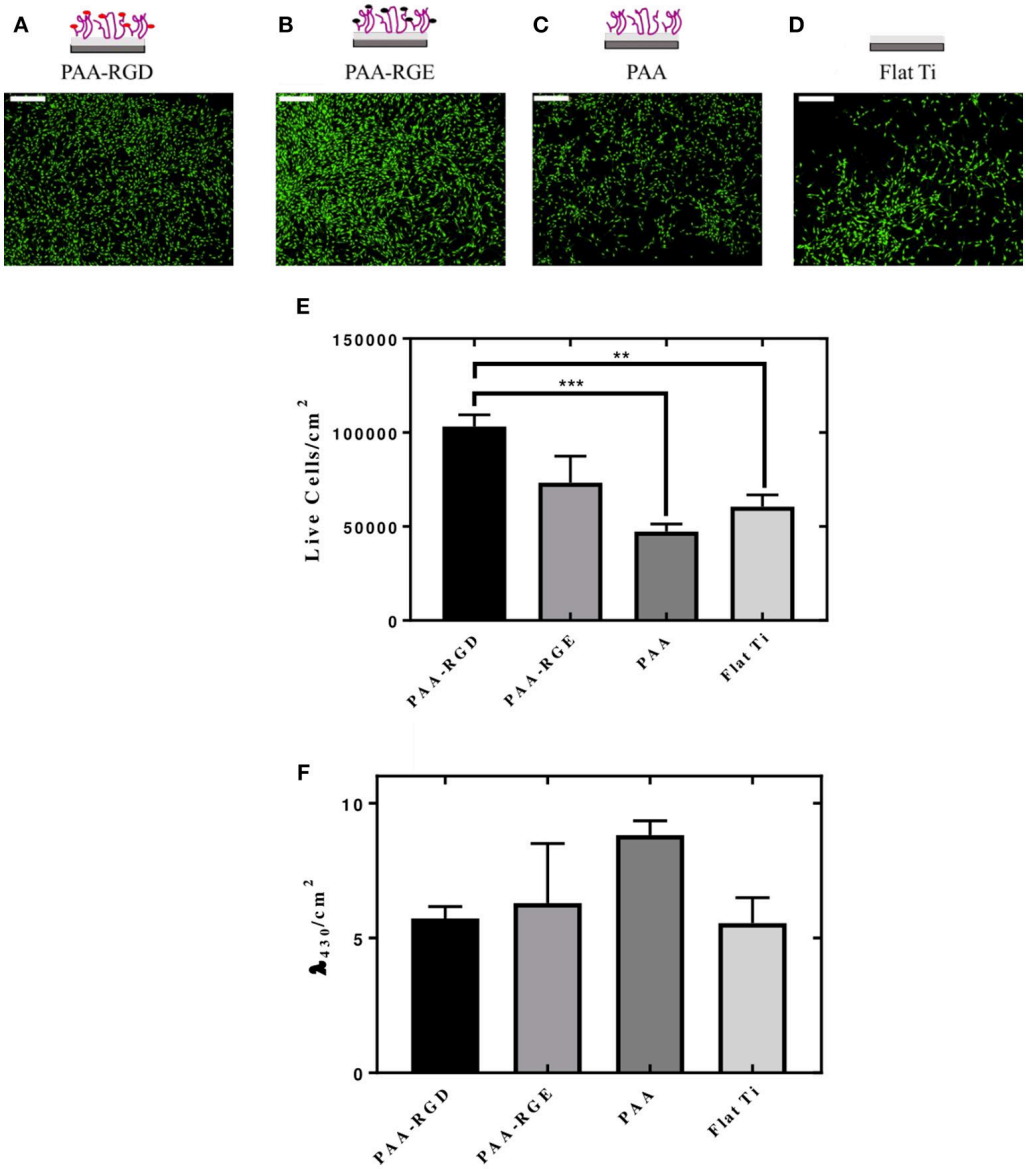
Adhesion and viability quantification of NIH/3T3 fibroblasts cultured on PAA brushes with bPEI-DNA complexes. Measurements of the adhesion and viability of NIH/3T3 mouse fibroblasts were acquired using calcein staining and water soluble tetrazolium (WST-1), respectively, with cells cultured on PAA brushes with bPEI-DNA complexes immobilized to the substrate, 48 h following cell seeding. For assessment of adhesion, cells were stained with calcein (2 μM) for 15 min before imaging. Cells cultured on all substrates exhibited healthy spreading and morphologies, as seen in representative images for PAA-RGD **(A)**, PAA-RGE **(B)**, PAA **(C)**, and Flat Ti **(D)** (Scale bar = 200 μm). Images were quantified for the live cells per area (cm^2^) using NIH ImageJ Processing Software. Statistical analysis was performed using one-way ANOVA with Tukey's post-tests, which showed a statistically significant difference between the number of live cells/cm^2^ on PAA-RGD compared to those on PAA (^***^P ≤ 0.001) and Flat Ti (^**^P ≤ 0.01) **(E)**. WST-1 quantification of cell viability after 48 hours was measured at an absorbance of λ = 430 nm and normalized to the area (cm^2^) and statistical analysis using a one-way ANOVA with Tukey's post-tests showed no statistical differences **(F)**.

The cellular responses of NIH/3T3 fibroblasts cultured onto PAA brushes with immobilized bPEI-DNA complexes were assessed, including the number of cells adhered per area (cm^2^) and cellular morphology. Morphologically, the cells were spread with filamentous extensions characteristic of fibrotic cells on all substrates investigated (Figures 5A–D). No evidence of cytotoxicity was visually detected from these investigations. The number of live cells per area (cm^2^) was higher on PAA-RGD compared to all other surfaces, which was significant compared to the number of cells adhered to PAA (^***^P ≤ 0.001) and Flat Ti (^**^P ≤ 0.01) (Figure 5E). Cell viability assays were performed in cells cultured on PAA-RGD, PAA-RGE, PAA, and Ti substrates with immobilized complexes (N/P 20), which showed no statistical differences in the viability of cells cultured on PAA-RGD, PAA-RGE, PAA, or Flat Ti (Figure 5F) after 48 h.

### Investigating the Effect of Free bPEI on Substrate Mediated Gene Delivery

**Figure 6:**
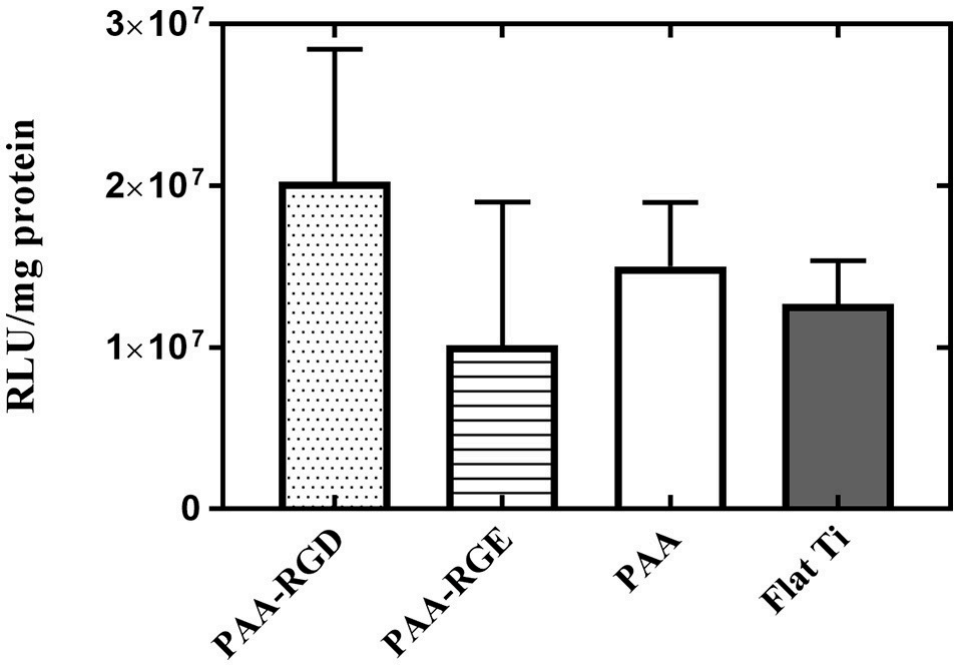
Substrate-mediated gene delivery of filtered bPEI-DNA complexes in NIH/3T3 fibroblasts. Fibroblasts were cultured onto filtered bPEI-DNA complexes immobilized onto the substrate for transfection. Filtered samples were centrifuged through a Vivaspin6 filter to remove free bPEI and the complexes were eluted from the filter. SMD studies were analyzed using one-way ANOVA with Tukey's post-test, and the results showed no significant difference between transfection success in cells cultured on immobilized filtered complexes on any substrate.

Given that DNA adsorption studies suggested that all surfaces loaded the same amount of DNA and ellipsometric measurements suggested there was additional organic matter (i.e., free bPEI) adsorbed to the substrates with PAA brushes, the role of free bPEI on SMD on polymer brush-modified substrates was investigated. To study the effect of free bPEI on transfection, free (i.e., uncomplexed) bPEI was filtered out from the formed bPEI-DNA complexes prior to immobilization to substrates for SMD. After the removal of free bPEI, there were no significant differences in transfection for NIH/3T3 fibroblasts cultured on any of the substrates (Figure 6). Furthermore, comparing the results of transfection using complexes (formed at N/P 20) with or without free bPEI (Figure 2D vs. Figure 6) showed that transfection mediated by filtered complexes was nearly two orders of magnitude lower than transfection mediated by unfiltered complexes with the free bPEI. Furthermore, similar to the cell viability measured on substrates with immobilized (unfiltered) complexes (Figure 5F), there were no statistical differences in the viability of fibroblasts cultured on PAA-RGD, PAA-RGE, PAA, or Flat Ti with immobilized filtered complexes (Supplementary Figure 2).

**Figure 7:**
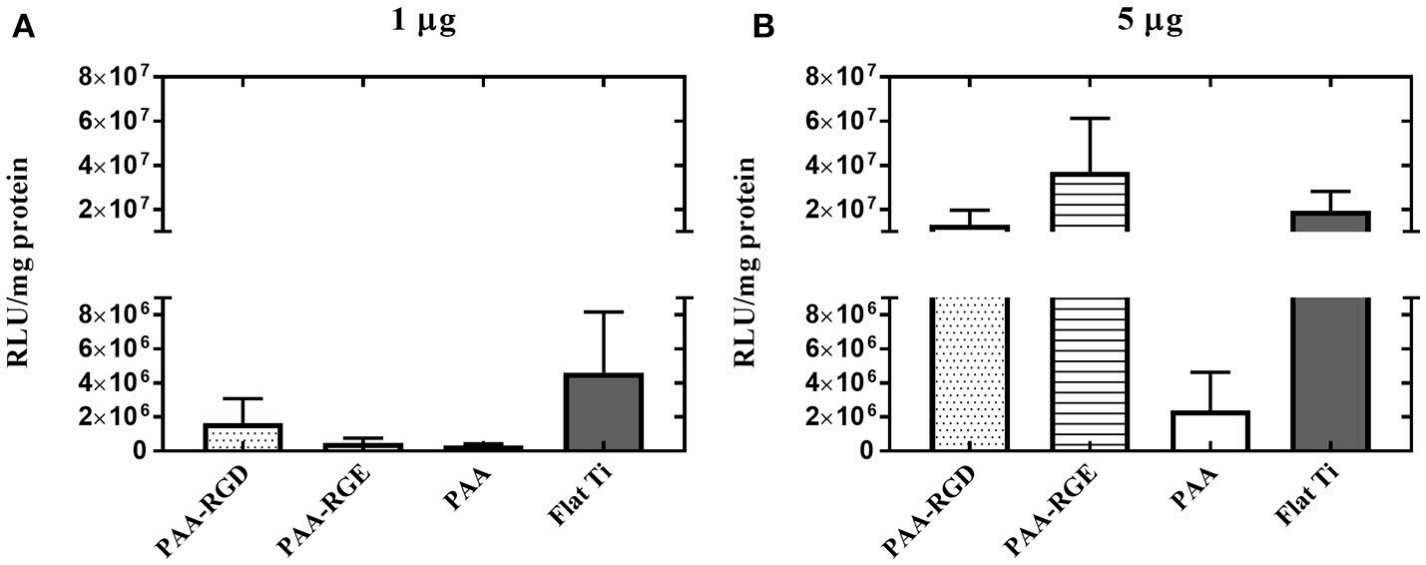
Substrate-mediated gene delivery of filtered bPEI-DNA complexes in NIH/3T3 fibroblasts with the addition of free bPEI. Fibroblasts were cultured onto filtered bPEI-DNA complexes immobilized to the substrate for transfection. At the time of complex immobilization, 1 **(A)** or 5 **(B)** μg of bPEI (1 μg/μL) was added concurrently to the substrates. SMD studies were analyzed using one-way ANOVA with Tukey's post-test, and there was no significant difference between transfection success in cells cultured with additional 1 or 5 μg free PEI.

To further elucidate the effect of free bPEI on transfection success, the addition of free bPEI was controlled by adding free bPEI to filtered complexes during immobilization to the different substrates. Two different amounts of free bPEI (1 or 5 μg) were added onto the substrate with the filtered complexes and immobilization was allowed to proceed for 2 h as described above. By defining the mass of DNA required in the final complex solution, the desired N/P ratio, and using the molecular weight of bPEI and the pEGFP-LUC plasmid, we were able to calculate the approximate masses of bPEI needed to form complexes at various N/P ratios. Furthermore, based on previous literature suggesting an N/P ratio of 3 results in fully complexed DNA with little to no excess free bPEI (Yue et al., 2011a), we were able to estimate the mass of complexed and free PEI present in the complex solution when forming complexes at varying N/P ratios. Using these calculations, the dose of free bPEI added was determined by subtracting the calculated mass of complexed bPEI required to complex 0.05 μg DNA (0.13 μg) from the calculated total mass of bPEI required (0.89 μg) for complexes formed at a N/P ratio of 20. Additionally, the calculated difference between the total mass immobilized to PAA(0.93 μg) and PAA-RGD (0.97 μg), both measured by ellipsometry (Figure 3B), and the mass accounted for by the complexed DNA and PEI (0.07 μg, calculated based on the mass of radiolabeled DNA measured plus the mass of PEI required to fully complex that mass of DNA) suggests an amount of ~1 μg of free PEI in solution (0.86 and 0.90 μg, respectively; Figure 3). For all substrates, although there were no statistical differences, increasing the amount of free bPEI increased the normalized transgene expression in a dose-dependent manner by one order of magnitude in cells cultured on substrates dosed with 5 μg compared to those dosed with 1 μg (Figure 7B vs. Figure 7A), except those on PAA. When investigating the substrate response by dose, there was no significant difference in transfection success for cells cultured on all substrates dosed with 1 μg of free bPEI (Figure 7A), but substrates dosed with 5 μg of free bPEI showed transfection one order of magnitude higher for cells cultured on PAA-RGD, PAA-RGE, and Flat Ti when compared to those on PAA (Figure 7B).

## Discussion

The objective of this work was to investigate the immobilization of DNA complexes to substrates functionalized with polymer brushes, taking advantage of the high negative surface charge of the brushes to attract and load cationic complexes, while also presenting cell-binding ligands to potentially influence the cellular response. Previous studies have indicated that the chemical properties of the substrate (e.g., self-assembly monolayers, polymer films, protein coatings) affect DNA complex binding and the efficiency of SMD (Segura et al., 2003; Bengali et al., 2005, 2007, 2009; Pannier et al., 2005, 2008; Li et al., 2009; Rea et al., 2009b; Zhang et al., 2009; Holmes and Tabrizian, 2013; Hu and Zheng, 2015), but many of those studies have focused on substrates like TCPS or glass, rather than biomaterials with possible clinical applications such as Ti. In this paper, we investigated the ability of chemically modified Ti substrates (with PAA brushes with or without peptide modifications) to support SMD. Building off our previous work, where we showed that PAA brushes grafted to Ti maintain swelling functionality and the addition of the RGD peptide enhances cell attachment compared to unmodified PAA (Rosenthal et al., 2018), herein we hypothesized that the highly negative charge of PAA (Psarra et al., 2015b) could allow for improved DNA complex adsorption and that cells cultured on PAA-RGD would have increased transfection success with SMD.

After determining that brushes were grafted and modified similarly to our previous investigation (Tables 1–3) (Rosenthal et al., 2018), we investigated the ability of PAA-RGD brushes to support SMD in NIH/3T3 fibroblasts. Fibroblasts were chosen given their frequent use in transfection studies (Rea et al., 2009a; Ryoo et al., 2010; Blocker et al., 2011; Kasputis and Pannier, 2012), and their role in wound healing. Fibroblasts cultured on PAA-RGD with immobilized bPEI-DNA complexes (for N/P ratios of 5, 10, 20) had the highest transfection compared to cells cultured on all other surfaces (PAA-RGE, PAA, Flat Ti). Transfection was significantly increased in cells cultured on PAA-RGD compared to cells cultured on PAA on surfaces when complexes formed at N/P 10 and N/P 20 were immobilized (Figure 2); complexes formed at these ratios exhibited the smallest diameters and highest positive charges (Supplementary Figure 1), two attributes that have previously been shown to produce high transfection success (Kunath et al., 2003). Using the highest N/P ratio, experiments were then performed to investigate the amount of DNA immobilized on and released from the substrate to determine if enhanced SMD on PAA-RGD substrates could be attributed to increased DNA adsorption (and thus dose presented to the cells), which is often what contributes to improved transfection success seen in SMD (Kasputis et al., 2016). Given that complexes formed at a N/P ratio of 20 exhibited the highest overall positive charge compared to those formed at lower N/P ratios (Supplementary Figure 1) and both PAA brushes (Psarra et al., 2015b) and the RGD peptide GRGDS (Psarra et al., 2017) have a negative charge under physiological conditions (pH 7.2; Figure 1), we hypothesized that PAA brushes would increase the amount of DNA loading. However, there was no increase in the amount of radiolabeled DNA immobilized onto PAA and PAA-RGD substrates compared to Flat Ti (Figure 3A). Furthermore, the amount of immobilized radiolabeled DNA measured on PAA substrates was within the range, albeit low, of previously reported studies using other substrates for SMD (Segura et al., 2003; Bengali et al., 2005; Pannier et al., 2005, 2008; Holmes and Tabrizian, 2013), further suggesting that PAA brushes do not increase the DNA loading capacity of the substrate. After analyzing the immobilization of complexes with radiolabeled DNA onto PAA, PAA-RGD, and Flat Ti substrates, the release of DNA was similarly measured with radioactivity using three different media conditions (OptiMEM, growth media, and conditioned media) to investigate the effect of electrostatics and competitive protein binding on the release of DNA from PAA brushes. The release profiles here are comparable to previously reported studies (Bengali et al., 2005; Pannier et al., 2005, 2008; Zhang et al., 2009; Holmes and Tabrizian, 2013), suggesting that the brushes provide sufficient release for transfection success. Three release media were used with different amounts of serum components (OptiMEM < growth culture media < conditioned media) and cellular metabolites (i.e., conditioned media) that aid release (Pannier et al., 2008). We hypothesized the amount of DNA released should correlate to the respective increase in serum/metabolites, yet the release profiles were similar regardless of release media (Figure 4), suggesting that the combined effect of competitive protein binding and electrostatics were similar for all media types. In addition, within each media condition, while there were some significant differences in release profiles among the different substrates, it is unlikely that the difference in the amount of DNA released accounted for the difference in transfection outcomes seen in Figure 2D.

Finally, cellular adhesion and viability in the presence of immobilized complexes on the substrates were investigated, as these are cellular behaviors known to influence transfection success (Pannier et al., 2005, 2008; Kasputis and Pannier, 2012). Cellular adhesion was enhanced significantly in cells cultured on PAA-RGD compared to those cultured on PAA and Flat Ti (Figure 5), which confirms results from our previous work (Rosenthal et al., 2018) and is expected due to the known effect of RGD on cell adhesion (Hersel et al., 2003). However, it should be noted that in comparison to cellular adhesion on these substrates without complexes (Rosenthal et al., 2018), cell adhesion was increased in this work on all substrates (i.e., PAA-RGD, PAA-RGE, PAA, Flat Ti) with immobilized complexes (Figures 5A–D), suggesting that immobilized bPEI-DNA complexes can increase cellular adhesion, even on nonfouling substrates (i.e., PAA-RGE, PAA). Similar observations have been made on other nonfouling substrates used for SMD (Pannier et al., 2008), and the promotion of cell adhesion on immobilized complexes has been attributed to possible interactions between serum proteins and the immobilized positively charged complexes (Pannier et al., 2008), which subsequently can promote adhesion. Along with the increase in positive charge of the substrate by the cationic complexes, the addition of peptides has been shown to alter the charge of the substrate (Psarra et al., 2017), which also may explain the similarity in cell adhesion on PAA-RGD and PAA-RGE substrates (Figure 5E), due to increased protein adsorption to the complexes immobilized on both substrates, allowing for cell adhesion.

Although complex immobilization significantly increased the number of adhered cells onto PAA-RGD compared to PAA and Flat Ti (Figure 5E), there was no significant difference in the viability of cells cultured on PAA-RGD, PAA-RGE, PAA, or Flat Ti (Figure 5F). While the presence of RGD was shown to improve cell adhesion and transfection, cell viability was shown to be similar and high on all substrates (Figure 5F). Many previous investigations of RGD-modified substrates have shown that cellular adhesion and viability are often related (Nuttelman et al., 2005; Salinas and Anseth, 2008; Bacakova et al., 2011). However, the difference between the results for adhesion and viability assays reported here is presumably due to the processing required for each technique, as adhesion staining has more wash steps in comparison to the WST-1 assay, which presumably results in only the most adhered cells remaining for image analysis. Like the investigations of DNA immobilization and release, the investigations of the cellular response also do not sufficiently explain the difference in transfection outcomes seen in Figure 2D.

Given that traditional indicators of successful SMD transfection (DNA immobilization and DNA release from the substrate, and the cellular response) did not explain the differences seen in SMD transfection among the different substrates, and adsorption measurements made using radiolabeled DNA only account for the mass of DNA adsorbed to the substrates (i.e., bPEI cannot be accounted for using radiolabeled DNA), we explored ellipsometric methods to measure and model the total amount of adsorbed mass (DNA and bPEI, both free and complexed). Using ellipsometry we showed that there was a significant increase in total mass immobilized onto the substrates modified with PAA and PAA-RGD compared to the mass on Flat Ti (Figure 3B). Given that it requires ~0.02 μg of 25 kDA bPEI to fully complex 0.05 μg DNA (based on the calculations as described above using the molecular weight of bPEI and DNA and N/P ratio of 3), which would result in a theoretical total mass of 0.07 μg for the fully formed complexes used in the adsorption studies (Figure 3B, dotted line), and there was nearly no difference in the amount of DNA measured on Flat Ti (Figure 3A) and total mass measured on Flat Ti (Figure 3B); we hypothesize ellipsometric measurements are underestimating the total mass on the Flat Ti, which has been shown in previous investigations with complex immobilization monitored by ellipsometry (Kasputis et al., 2014). However, even with underestimation, the increased mass measured on PAA-RGD and PAA compared to Flat Ti is large, and may be from the adsorption of complexed bPEI but also free bPEI, as free bPEI in the complexing solution has been previously suggested as a component of the immobilized material in SMD (Pannier et al., 2008). Based on the assumptions that a N/P of 3 will have no free bPEI (Yue et al., 2011a) and calculations to determine the polymer present in a solution formed for complexes at an N/P of 20, we estimate a mass of approximately 0.76 μg of free bPEI was present in the complex solution used for taking ellipsometric measurements, which is similar to the change in mass for substrates modified with PAA and PAA-RGD compared to Flat Ti (0.86 and 0.90 μg, respectively; Figure 3).

In bolus studies, free bPEI has been proposed to increase overall gene transfection efficiency by up to hundreds of fold (Boeckle et al., 2004; Deng et al., 2009; Dai et al., 2011; Yue et al., 2011a,b; Bonner et al., 2013). Specifically, free bPEI has been suggested to reduce charge interactions that repeal complexes from the cellular membrane, reduce lysosomal entrapment of complexes, assist translocation of complexes through the nuclear membrane, enhance transcription, and facilitate translocation of mRNA (Cai et al., 2016). The role of free bPEI has not been significantly investigated for SMD, given that traditional SMD methods usually perform a rinse after immobilization of DNA complexes to remove loosely bound complexes (Pannier et al., 2005, 2008; Bengali et al., 2009). Therefore, rinsing the substrates would presumably result in free bPEI also being washed away from the surface before performing SMD, as seen on bare Flat Ti in this current study (Figure 3B). However, the highly negative PAA brushes could allow for the capture of the positively charged free bPEI to the substrates, which may improve subsequent transfection. Therefore, we hypothesized that the increase in transfection seen in cells cultured on bPEI-DNA complexes immobilized to PAA-RGD may be related to free bPEI attracted to the brushes. To test this, we investigated the effect of free bPEI on transfection success by performing transfection with filtered complexes (i.e., free bPEI removed) and complexes formed with different N/P ratios to tune the amount of free bPEI in the complexing solution, which has previously been shown to dramatically affect transfection success (Dai et al., 2011; Bonner et al., 2013). The removal of all free bPEI through a size-exclusion membrane resulted in a substantial decrease in transfection by two orders of magnitude compared to transfection performed with unfiltered complexes (Figure 6 vs. Figure 2) and transfection was not different amongst the investigated substrates (Figure 6), which supported our hypothesis that the presence of free bPEI may enhance transfection. These results are similar to those for bolus delivery studies that show the presence of free bPEI enhances transfection success (Boeckle et al., 2004; Deng et al., 2009; Dai et al., 2011; Yue et al., 2011a,b; Bonner et al., 2013), thereby suggesting that free bPEI could also enhance transfection success in SMD on PAA-RGD, possibly through bPEI adsorption and subsequent release from the PAA-RGD surface. To further investigate the role of free bPEI in SMD, investigations were performed using the filtered complexes immobilized to the substrate, but with the addition of free bPEI (1 or 5 μg) to the complexing solution during immobilization. As previously stated, the doses of free bPEI were determined by the estimated amount of free bPEI in the complexing solution, which was calculated to be about 0.76 μg, and the difference in mass calculated for substrates modified with PAA and PAA-RGD compared to Flat Ti (0.86 and 0.90 μg, respectively; Figure 3). Therefore, a dose close to the calculated amount (1 μg) and a dosage in excess (5 μg) were chosen as free bPEI amounts to immobilize with filtered complexes. Transfection outcomes were then assessed, which showed an increase in transfection success for all surfaces, except for PAA, in a dose-dependent manner (Figure 7), further validating the importance of free bPEI for enhancing transfection. In addition to studies with filtered complexes, the dose of free bPEI can also be controlled simply by forming complexes at various N/P ratios. Complexes formed at a N/P of 3 have been shown to have little to no free bPEI (Yue et al., 2011a) and showed low SMD transfection success in our investigation. Conversely, complexes at higher ratios (i.e., 5, 10, 20) have been shown to have more free bPEI (Dai et al., 2011), and in our investigations showed an increase in transfection levels that corresponded with the increase of the N/P ratio, thereby supporting our hypothesis that transfection is influenced by the presence of free bPEI on the substrates. Furthermore, viability was also studied on substrates with immobilized filtered complexes (Supplementary Figure 2), which showed, like in viability assays on substrates with immobilized unfiltered complexes (Figure 5F), that there was no statistical difference in viability as a function of substrate modification. More importantly, cell viability was not statistically different on filtered complexes (Supplemental Figure 2) compared to unfiltered complexes (Figure 5F), which suggests that free bPEI (which is present in unfiltered complexes immobilized on substrates in Figure 5F), does not negatively impact the cellular response to the substrate.

Finally, in addition to free bPEI, the RGD ligand on PAA-RGD may be aiding SMD transfection success with complexes at higher N/P ratios (Figures 2B–D), given the transfection was enhanced in cells cultured on complexes immobilized to PAA-RGD substrates compared to PAA-RGE. Fibroblasts (e.g., the NIH/3T3 cell line) are known to express integrin α5β1 (Dalton et al., 1995), which is known to aid cell adhesion through binding to RGD (Massia and Hubbell, 1991; Suehiro et al., 2000; Humphries et al., 2006), and supports the results of our previous work (Rosenthal et al., 2018) and work shown here (Figure 5E) that show an increased number of cell attached to PAA-RGD compared to the control surfaces. Furthermore, the inclusion of the RGD ligand may activate signaling cascades that regulate cell processes pivotal for transfection, such as endocytosis and internalization (Hersel et al., 2003; Garcia, 2005). Integrin binding to RGD ligands has been shown to improve bolus nonviral gene delivery (Kong et al., 2007) and SMD (Rea et al., 2009b), via the RGD motif on fibronectin coatings for both types of delivery (Bengali et al., 2007; Dhaliwal et al., 2010, 2012). However, the role of the RGD ligand in our system here requires further investigations to understand its role in transfection success.

## Conclusions

In our previous study, we showed that PAA brushes can be “grafted-to” Ti substrates and RGD can be conjugated to these brushes to support cell adhesion (Rosenthal et al., 2018). Herein, we investigated those PAA-RGD modified Ti substrates as a platform for improving SMD to NIH/3T3 fibroblasts using immobilized bPEI-DNA complexes. From our studies, we found that transfection was significantly increased on PAA-RGD modified substrates, but this improvement in transfection could not be attributed to the amount of DNA immobilized to the surface or the DNA release profile. Instead, we found that substrates modified with PAA brushes adsorb more overall mass, which may be attributed to immobilization of free and complexed bPEI, as measured with spectroscopic ellipsometry. To confirm the role of free bPEI in SMD on PAA-RGD substrates, transfection investigations were performed with filtered complexes and controlled dosages of free bPEI. The results of these transfection investigations with filtered complexes suggest that free bPEI is beneficial to transfection success and PAA brushes allow for the adsorption and presentation of free bPEI in a SMD format. To our knowledge, this paper is one of the first reports using polymer brushes grafted to a Ti substrate for SMD and the conclusions from our findings suggest that these substrates can enhance the cellular response to transfection via SMD. Therefore, future studies will investigate the adjuvant-like effect of free bPEI in cells cultured on PAA-RGD brush substrates through further optimization of the dosage and complex formation, as well as investigations into the intracellular mechanisms affected by RGD and free bPEI that are involved in transfection efficiency (i.e., endocytosis, trafficking). Overall, the findings of this article suggest that the modification of Ti with PAA-RGD may be a future platform for applications that could be improved by gene delivery such as biomedical devices, implantable sensors, and diagnostics tools.

## Author Contributions

AM and AR made substantial contributions to the design, acquisition, analysis, and interpretation of data, and participated in drafting the article. EF made substantial contributions to the acquisition and interpretation of data, and participated in drafting the article. TK made substantial contributions to the interpretation of data and participated in drafting the article. EB made substantial contributions to the analysis and interpretation of data and participated in drafting the article. SN made substantial contributions to the acquisition of data and substrate preparation. ES and MatS made substantial contributions to the design and analysis of data. ManS participated in drafting the article. AP and PU made substantial contributions to the design, acquisition, analysis, and interpretation of data, participated in drafting the article and provided funding. All authors gave approved the final version of the manuscript.

### Conflict of Interest Statement

The authors declare that the research was conducted in the absence of any commercial or financial relationships that could be construed as a potential conflict of interest.

## Supplementary Material

Supplementary Figure 1 Zeta potential and sizing of bPEI-DNA complexes with varied N/P ratios. bPEI-DNA complexes were formed with 2 μg of DNA at N/P of 3, 5, 10, or 20, and the zeta potential **(A)** and size **(B)** of the complexes were determined by dynamic light scattering and Laser Doppler micro-electrophoresis, respectively, at room temperature. Statistical differences between the measurements for zeta potential (and complex diameter) were analyzed using one-way ANOVA with Tukey's post-test. The zeta potential measurements **(A)** showed a significant increase in the charge of all complexes formed at the higher N/P ratios (5, 10, and 20) compared to those formed at N/P of 3 (^****^*P* ≤ 0.0001), as well as a significant increase in the charge of complexes formed at N/P of 10 and 20, compared to those formed at a N/P of 5 (^*^*P* < 0.05, and ^**^*P* ≤ 0.01, respectively). The sizing of the complexes **(B)** showed that complexes formed at a N/P ratio of 3 were significantly larger than those formed at all other N/P ratios (5, 10, and 20) (^***^*P* ≤ 0.001, ^**^*P* ≤ 0.01, and ^***^*P* ≤ 0.001 respectively), and complexes formed at a N/P ratio of 20 were significantly smaller than those formed at a N/P of 5 and 10 (^****^*P* ≤ 0.0001).

Supplementary Figure 2 Viability quantification of NIH/3T3 fibroblasts cultured on PAA brushes with filtered bPEI-DNA complexes. The measurement of the viability of NIH/3T3 mouse fibroblasts was acquired using water soluble tetrazolium (WST-1) with cells cultured on PAA brushes with filtered bPEI-DNA complexes immobilized to the substrate, 48 h following cell seeding. WST-1 quantification of cell viability was measured at an absorbance of λ = 430 nm and normalized to the area (cm^2^). Statistical analysis was performed using one-way ANOVA with Tukey's post-tests, which showed no statistical differences.
