# Supplementary figures and images for: Free Polyethylenimine Enhances Substrate-Mediated Gene Delivery on Titanium Substrates Modified With RGD-Functionalized Poly(acrylic acid) Brushes
Source: Front Chem. 2019 Feb 7;7:51. doi: 10.3389/fchem.2019.00051 (PMC6374293; doi:10.3389/fchem.2019.00051)

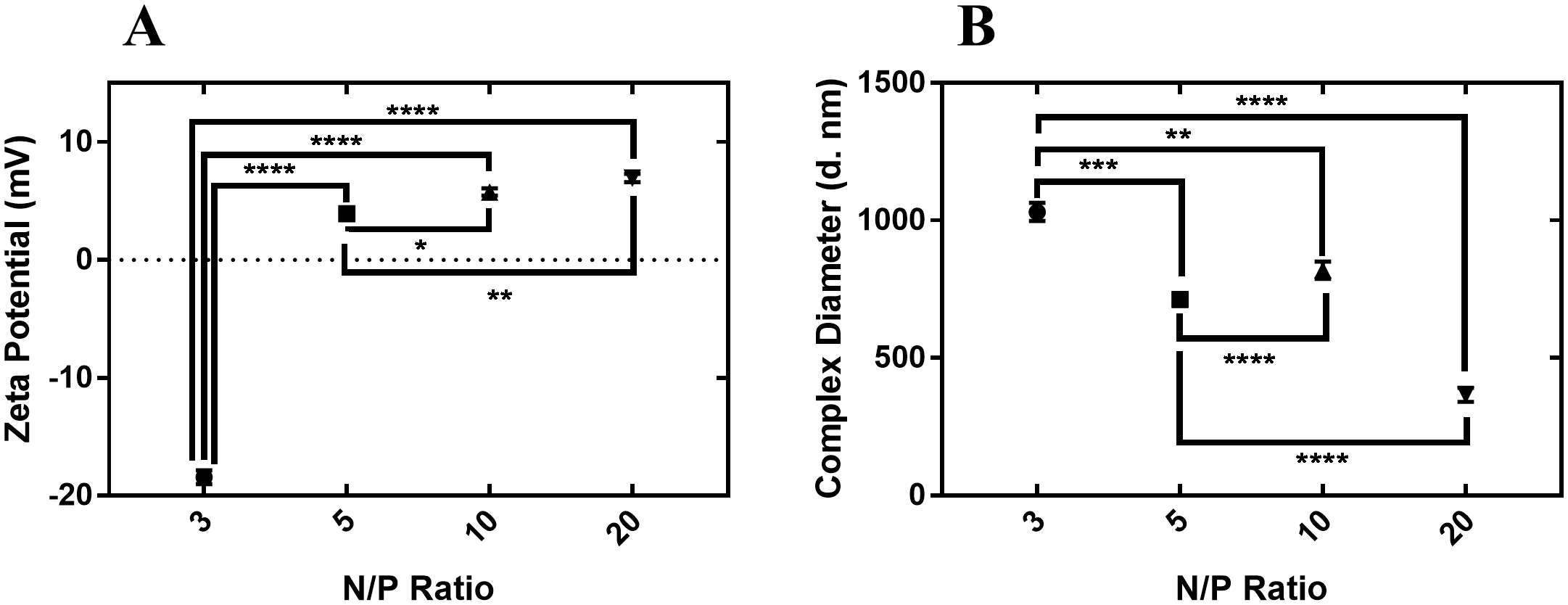

Supplement: Supplementary Figure 1 — Zeta potential and sizing of bPEI-DNA complexes with varied N/P ratios. bPEI-DNA complexes were formed with 2 μg of DNA at N/P of 3, 5, 10, or 20, and the zeta potential (A) and size (B) of the complexes were determined by dynamic light scattering and Laser Doppler micro-electrophoresis, respectively, at room temperature. Statistical differences between the measurements for zeta potential (and complex diameter) were analyzed using one-way ANOVA with Tukey's post-test. The zeta potential measurements (A) showed a significant increase in the charge of all complexes formed at the higher N/P ratios (5, 10, and 20) compared to those formed at N/P of 3 (****P ≤ 0.0001), as well as a significant increase in the charge of complexes formed at N/P of 10 and 20, compared to those formed at a N/P of 5 (*P < 0.05, and **P ≤ 0.01, respectively). The sizing of the complexes (B) showed that complexes formed at a N/P ratio of 3 were significantly larger than those formed at all other N/P ratios (5, 10, and 20) (***P ≤ 0.001, **P ≤ 0.01, and ***P ≤ 0.001 respectively), and complexes formed at a N/P ratio of 20 were significantly smaller than those formed at a N/P of 5 and 10 (****P ≤ 0.0001). [file Image_1.TIF]

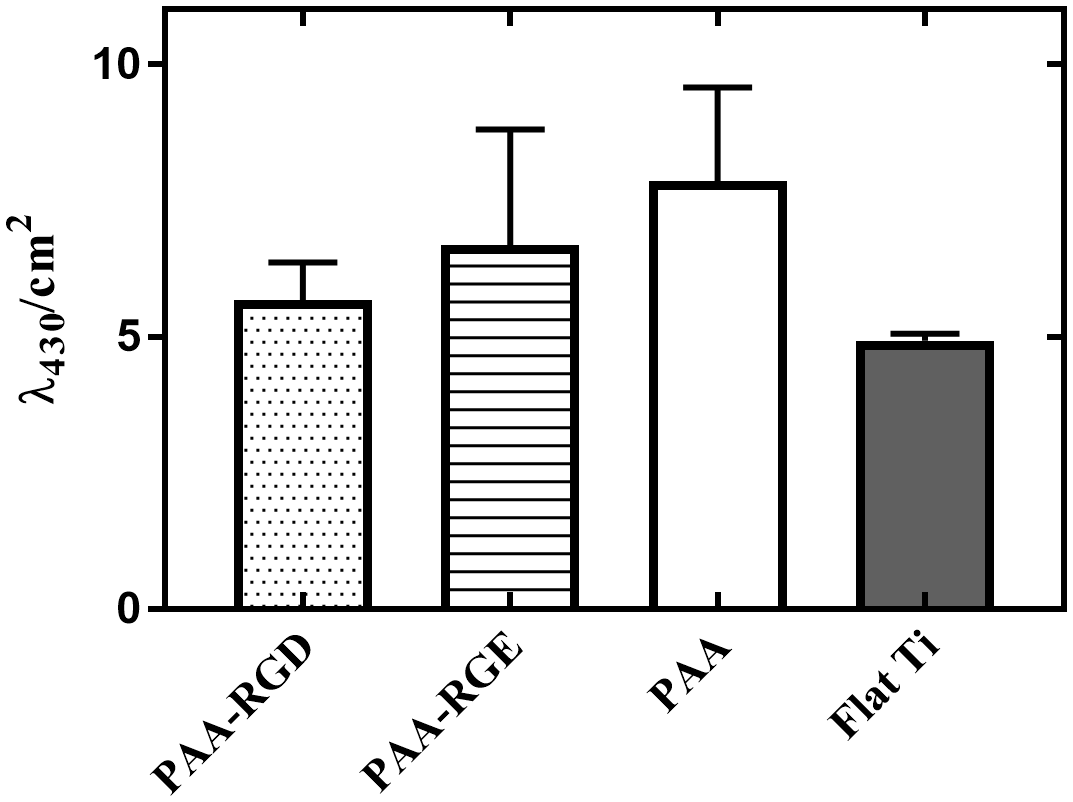

Supplement: Supplementary Figure 2 — Viability quantification of NIH/3T3 fibroblasts cultured on PAA brushes with filtered bPEI-DNA complexes. The measurement of the viability of NIH/3T3 mouse fibroblasts was acquired using water soluble tetrazolium (WST-1) with cells cultured on PAA brushes with filtered bPEI-DNA complexes immobilized to the substrate, 48 h following cell seeding. WST-1 quantification of cell viability was measured at an absorbance of λ = 430 nm and normalized to the area (cm2). Statistical analysis was performed using one-way ANOVA with Tukey's post-tests, which showed no statistical differences. [file Image_2.TIF]
